# Rationale, design and methods for a randomised and controlled trial to evaluate "Animal Fun" - a program designed to enhance physical and mental health in young children

**DOI:** 10.1186/1471-2431-10-78

**Published:** 2010-11-04

**Authors:** Jan P Piek, Leon M Straker, Lynn Jensen, Alma Dender, Nicholas C Barrett, Sue McLaren, Clare Roberts, Carly Reid, Rosie Rooney, Tanya Packer, Greer Bradbury, Sharon Elsley

**Affiliations:** 1 School of Psychology and Speech Pathology, Curtin University, Perth, Australia; 2 School of Physiotherapy, Curtin University, Perth, Australia; 3 School of Occupational Therapy and Social Work, Curtin University, Perth, Australia; 4 Curtin Health Innovation Research Institute, Curtin University, Perth, Australia

## Abstract

**Background:**

Children with poor motor ability have been found to engage less in physical activities than other children, and a lack of physical activity has been linked to problems such as obesity, lowered bone mineral density and cardiovascular risk factors. Furthermore, if children are confident with their fine and gross motor skills, they are more likely to engage in physical activities such as sports, crafts, dancing and other physical activity programs outside of the school curriculum which are important activities for psychosocial development. The primary objective of this project is to comprehensively evaluate a whole of class physical activity program called *Animal Fun *designed for Pre-Primary children. This program was designed to improve the child's movement skills, both fine and gross, and their perceptions of their movement ability, promote appropriate social skills and improve social-emotional development.

**Methods:**

The proposed randomized and controlled trial uses a multivariate nested cohort design to examine the physical (motor coordination) and psychosocial (self perceptions, anxiety, social competence) outcomes of the program. The *Animal Fun *program is a teacher delivered universal program incorporating animal actions to facilitate motor skill and social skill acquisition and practice. Pre-intervention scores on motor and psychosocial variables for six control schools and six intervention schools will be compared with post-intervention scores (end of Pre-Primary year) and scores taken 12 months later after the children's transition to primary school Year 1. 520 children aged 4.5 to 6 years will be recruited and it is anticipated that 360 children will be retained to the 1 year follow-up. There will be equal numbers of boys and girls.

**Discussion:**

If this program is found to improve the child's motor and psychosocial skills, this will assist in the child's transition into the first year of school. As a result of these changes, it is anticipated that children will have greater enjoyment participating in physical activities which will further promote long term physical and mental health.

**Trial registration:**

This trial is registered in the Australian and New Zealand Clinical trials Registry (ACTRN12609000869279).

## Background

Movement in children is critical to physical, mental, and emotional adjustment in childhood and adolescence [1]. Key child health issues of international importance include childhood obesity, and its serious health implications, and mental health issues such as anxiety and depression.

The world prevalence of overweight in school-aged children has been estimated at 10% with 25% of these being obese [2]. Furthermore, this proportion continues to rise, particularly in economically developed regions such as Canada and Australia. However, interventions targeting diet and/or physical activity have had limited success [3]. Furthermore, childhood overweight/obesity is not just associated with poor physical health but also poor psychosocial health, particularly in boys [4].

Anxiety and depression are the most common mental health problems experienced by Australian children and adolescents [5], and are significant problems worldwide [6]. Depressive disorders have been estimated to have a lifetime prevalence in adolescence of between 15 - 20% and 6 month prevalence in adolescence of between 5 - 6% and between 1 - 3% in children [7,8]. The 12 month prevalence rates for anxiety disorders are approximately 8% in children and between 11% and 20% in adolescents [9]. Internalising problems such as anxiety and depression are particularly burdensome for children, interfering with social, cognitive, emotional, and academic life at a time when other children are building skills and competencies [10,11].

### Motor ability is linked with obesity and mental health

In addressing these two key childhood issues of obesity and poor mental health, an approach that has been overlooked is one which links these problems in early development, namely their relationship with motor ability.

It is well established that a lack of physical activity is considered a key factor underlying childhood obesity [12], and a lack of physical activity has been linked to other problems such as lower mineral density and cardiovascular risk factors [13]. Children with poor motor ability have been found to engage less in physical activities than other children [14]. To engage successfully in physical activities children need to have a certain degree of motor competence, and competence motivation theorists such as Harter [15] argue that confidence in his or her own ability to perform any activity partly determines the child's involvement level. If children are confident with their motor skills, they are more likely to engage in physical activities such as sports, dancing and other physical activity programs outside of the school curriculum [16]. Trost et al. [12] found that in sixth grade children, physical activity self-efficacy was a key variable which distinguished the obese and non-obese children. Previous research [1,17]has demonstrated that children with poor motor ability perceive themselves as less athletically competent, which has been linked to higher levels of anxiety [1] and depressive symptomatology [18].

Schoemaker and Kalverboer [19] established a link between motor coordination difficulties and social and affective problems in children as young as 6 years. Since then, research has identified that children with motor deficits have social and emotional problems such as poorer self-worth [1,20], and have higher levels of anxiety [1,21], and depression [18,22]. Piek, Bradbury et al.'s study [21] found that kindergarten children's level of motor coordination was negatively related to anxious/depressed behavior as reported by the mother, which is consistent with the finding for older children. However, this finding is of serious concern as these children were only 4-5 years of age. Furthermore, recent findings have shown that gross motor performance in infancy and early childhood predict later anxiety and depressive symptomatology in school age children [23], and if young school-age children have both motor and anxiety problems they are more likely to have psychosocial difficulties in adolescence [24].

### Motor ability is linked with other aspects of development

Until recently, there has been little evidence provided for a relationship between motor ability and other aspects of development such as cognitive and language development. However, recently, an association between early development of gross motor skills and later cognitive abilities in children [25] and adults [26] has been identified. Other research [27] has found that motor ability in children is related to cognitive, language and empathic ability. Children with poor motor ability have been found to perform more poorly on cognitive tasks such as working memory [25,28], and emotional recognition [29]. Poor motor ability is also associated with many of the major developmental disorders such as Attention Deficit Hyperactivity Disorder [30,31], language impairment [32], Reading Disability [33] and Autism [34]. Many of these disorders have been found to be comorbid with Developmental Coordination Disorder (DCD), recognised by the American Psychiatric Association through its inclusion in the DSM-III in 1987 and in subsequent editions [35]. Children with DCD have a significant impairment in their motor coordination that cannot be attributed to any neurological deficit.

A recent study by Bart, Hajami and Var-Haim [36] identified the importance of motor ability in kindergarten on the transition to school. They found that motor function at the kindergarten (equivalent to our Pre-Primary) stage was predictive of scholastic adaptation and social and emotional adjustment 12 months later in the child's first year of school. This finding is particularly relevant to the proposed project.

### Interventions to improve motor ability

Despite the importance of motor development, there has been surprisingly little research examining the most effective way of teaching motor skills in schools. A primary focus has been on physical activity and sport skills, but there has been less emphasis on appropriate skill development for everyday functioning, particularly fine motor ability that is crucial for skills such as hand-writing. There are many motor skills programs currently available, but there is little information on how effective they are, and few have been developed for the Pre-Primary age. The *Move it Groove it *program, based in the state of New South Wales in Australia, was developed for 8 to 10 year olds [37]. Based on the mastery of fundamental movement skills (FMS), the developers of this program argued that it produced substantial improvement in movement skills over a relatively short period of time. Another program that relies on the development of FMS is the *FMS program *[38] that has been adopted by the Department of Education in Western Australia. There are three categories of skills that are included, body management (4 skills); locomotor skills (9 skills) and object control (9 skills - all ball skills). The argument behind this approach is that fundamental skills such as running, throwing, skipping and balancing are the building blocks for improving more complex sports. As mentioned before, the focus is on sports and physical activity participation. The latter program has not had any published reviews of its efficacy. The *Sports Play and Recreation for Kids *program [39] was developed in the US for elementary students and has been evaluated. This program included classes taught by movement experts as well as others by trained teachers. Both were found to increase physical activity levels *in class*. They also demonstrated improved movement skills. However, in all of these programs there is a lack of emphasis on fine motor ability, and it is known that both fine and gross motor ability are important for appropriate skill and mental health development [40]. In particular, poor motor ability has been linked to poor visual-spatial organisation [41,42], and this component needs to be more seriously considered in early movement programs.

According to Trost et al. [43], the following are important to include in physical activity intervention programs for children:

(1) provide activities that are developmentally appropriate so children can succeed

(2) provide the opportunity to observe influential others such as parents and peers performing physical activity

(3) provide verbal encouragement to participate

(4) reduce or eliminate grading or competition in the activity in order to reduce anxiety.

Another important factor is to ensure that the program is inclusive, that is, adopting a universal program for all children within the class. By including all children it reduces the stigma associated with being placed into a 'special' program. Furthermore, given that socially disadvantaged children are generally at higher risk for physical and mental health problems [44], universal interventions with socially disadvantaged children are likely to help prevent a higher proportion of problems in these children. For sustainability and continuity to be maximised it has also been argued that community interventions based in the school or home environment are the most appropriate places for universal interventions to occur [45]. Including all children is a challenge, as it means it must be interesting and exciting for those students who are both physically and socially competent, while being easy enough for the students who are struggling.

In order to accommodate both these needs, the program needs to provide considerable flexibility. These were the key concerns when developing the *Animal Fun *program.

### The Animal Fun program

The *Animal Fun *program is a Pre-Primary program developed to promote motor and social development. It includes four modules focusing on gross motor development, four modules focusing on fine motor development, and a module on social/emotional development.

The gross motor modules are designed to promote good static and dynamic balance, increase strength in lower limb muscles, and to develop an interest in locomotor activities such as running, skipping, jumping, climbing and hopping, correct techniques for throwing, catching and kicking, and more complex movement based on combining movements together.

The fine motor modules include activities that develop postural stability, and strengthen shoulder, elbow, wrist and hand muscles. They are also designed to develop sequencing in fine motor activities, and promote pre-scissor and pre-writing skills. The more advanced skills involve tool manipulation and the development of successful, mature and functional use of pencils, scissors, keyboards, mouse and joystick.

The social/emotional module includes activities where children are taught to accurately identify, label and monitor their feelings [46]. It is based on the Aussie Optimism program (AOP) developed by Clare Roberts [47] and adapted for younger children by Rosie Rooney [48].The Aussie Optimism Feelings and Friends Program is a 10-module program that has been adapted for year 1 and 2 students as well as a version for slightly older students in year 3 whose reading and writing skills and emotional repertoire is more developed. The programs include cognitive and behavioural intervention strategies and targets social, emotional and cognitive risk and protective factors for anxiety and depression. In modifying this for Pre-Primary children, the *Animal Fun *program has focused on the modules of Laughter, Identifying and labeling feelings, Breathing, and Relaxation.

The movement components of the *Animal Fun *program were developed based on the principle that to be successful in movement activities, children need to have a degree of motor competence, plus confidence in their ability to perform the activity [49]. Appropriate technique is an important aspect of motor skill development. However, research demonstrates that if children enjoy movement activities then it is more likely that they will participate, and will begin to build skills [50]. Participation promotes social interaction but also leads to practice which is the key to improving skills. In other words, if the children enjoy what they are doing, they will practice it and will improve their skills. Another key principle is how meaningful it is to the child [50]. By imitating animals as part of the program, the child can attach meaning to the tasks which adds to the fun and enjoyment.

The *Animal Fun *program was developed in consultation with teachers, developmental and clinical psychologists, physiotherapists, occupational therapists, speech therapists and health professionals working in the field with children who have motor disorders. The process involved an 8 month initial development followed by a pilot study and subsequent program refinement.

The pilot study was conducted with 120 children from 3 schools in low socioeconomic areas, with one school having the intervention and the other two smaller schools as controls. Pre-testing occurred in school term 1, the *Animal Fun *program was delivered in school terms 2 and 3, and post-testing occurred in term 4. Both motor and psychosocial variables were recorded pre- and post- intervention. Given the short time-frame, improvement in motor development was not anticipated (and not found) at post-testing, although it was anticipated that it would be observed later in development, hence the need to follow the children's progress into Year 1.

However, there were very promising psychosocial findings. Teachers returned 87 *Social Skills Rating Scales (teacher version) *forms, and a MANCOVA including all three subtests (cooperation, self-control and assertion) gave a statistically significant time x group interaction (F(3, 81) = 10.363, p < .001; partial η^2 ^= .277). Univariate analyses showed that the intervention produced a significant improvement in cooperation (F(1,83) = 21.507, p < .001; partial η^2 ^= .206). Behavioural measures of internalising, externalising, problem behaviour and academic competence failed to reach significance but all showed improvements in the predicted direction for the intervention group. At the conclusion of the post-testing, a focus group was run to determine the teachers' views on the program. All teachers found it easy to incorporate into the curriculum, and all were positive about the program with some of the comments as follows: The program was "really fun -the kids love it"; "easy to use and easy to follow"; "flexible", "was very adaptable"; "very little preparation time"; "good range of difficulty levels"; "all children joined in" With pilot study results showing an improvement in social skills and greater power being needed to evaluate the other potentially beneficial effects of the program, a large randomised controlled trial evaluating the efficacy of *Animal Fun *is needed.

In addition to the importance of motor ability for physical activity and fine motor skills such as hand-writing, movement skills are also linked with social interaction and social-emotional adjustment. There is also evidence that the early years before formal school may be an important time to invest in motor development given its relationship with the transition to school [36]. Given the promising results provided by our pilot study for the *Animal Fun *program, the current study proposes to evaluate the program in a randomised control trial with a larger sample of Pre-Primary children in both metropolitan and regional schools, to determine the impact on motor and psychosocial development immediately after the intervention and then 12 months following intervention once the child has entered grade 1 of school.

## Methods/Design

### Design and Aims

This study will use a randomized, cluster controlled trial to examine the influence of a school-based movement and psychosocial program on the physical and mental health of young children in their first years of school. A multivariate nested cohort design [51] will be used to examine the physical (motor coordination; BMI) and psychosocial (self perceptions, anxiety, social competence) outcomes of the program at the end of the Pre-Primary year and also 1 year later after the transition to primary school. Pre-intervention scores on motor and psychosocial variables for both control and intervention schools will be compared with post-intervention scores and scores taken 12 months later in Year 1.

### Participants

Twelve government primary schools from both metropolitan and regional areas will be invited to participate in this project. It is anticipated that 8 schools will commence in the first year of the study with the remaining 4 the following year with half being allocated as intervention schools and the other as control schools. Schools will have 50 or more students (two or more classes) enrolled in Pre-Primary classes for children aged 4 or 5 years. Schools from low socio-economic areas with a decile ranking of 7 or above will be targeted as it is anticipated they will have higher rates of physical and mental problems [44]. Regional centres will be included as they have been identified as areas in need of assistance, particularly as there are limited resources available for children with movement and psychosocial problems, and high rates of mental health problems have been identified in these regions [52].

Allowing for 10% non-participation for the pre-intervention testing, we expect the first data collection to include 540 children (270 in the control schools and 270 in the intervention schools). As the sample will represent a selected group of children from low SES areas rather than a random sample for all socioeconomic groups, there is evidence that attrition and movement of students will be greater in this sample. Hence, we conservatively estimate that we will have lost 30% of students at the 12 month follow-up in Year 1 leaving 190 children in each of the groups (N = 380). This is approximately 30 children per school, which is double that required to have an 80% chance of detecting an effect for the movement variables in a nested design with 12 schools (approximately 15 students per cluster or N = 180).

This study will have a broad inclusion policy and all Pre-Primary children and their parents in the selected schools will be invited to participate in the project. Children from non English speaking backgrounds and those with diagnosed and undiagnosed disabilities or developmental delays will be included in the testing wherever possible and only omitted from the project if data collection is impossible. Children's ages will range from 4 years in the initial testing in Pre-Primary to 7 years in the final post test in Year 1.

School principals and parents will be provided with a detailed written description of the project: its purpose, procedures, risks and benefits and given an opportunity to ask research staff questions or seek clarification prior to signing assent (children) and consent (school principals and parents) to participate. Schools, parents and children will be advised that they are free to withdraw from the project at any time. This study has ethical approval from the Human Research Ethics Committee of Curtin University of Technology (approval number HR02/2009).

### Intervention and control condition

Participating schools will be randomly assigned using a toss of a coin to the control or intervention condition. Schools will be paired on SES ranking, location and size, with one of the pair being randomly allocated as a control school and the other as an intervention school. Apart from the 3 testing sessions, the control school will follow the normal curriculum.

Teachers from the Intervention schools will be provided with comprehensive training in the *Animal Fun *Program either at their school or at Curtin University with presentations from the lead investigators. Information will be provided not only on the actual implementation of the *Animal Fun *activities but on the background and rationale for the project. The implementation of the program will be monitored with regular visits from the Research Coordinator to provide support to teachers and to obtain their feedback regarding children's enthusiasm for the activities, appropriateness of activity level of difficulty ratings and to discuss any issues or obstacles to including the program in their daily curriculum.

Teachers will also be asked to complete a weekly dosage report indicating which of the *Animal Fun *activities were implemented into the curriculum for that week and an estimate of the total time spent on *Animal Fun *for the same time period.

#### Animal Fun Program

The *Animal Fun *program was developed as a Pre-Primary program to promote motor and social development. This is a universal program for all children within the class. By including all children it reduces the stigma associated with being placed into a 'special' program. In order to accommodate the needs of all children, the program was developed to provide considerable flexibility. It does not give set lessons but provides information on the difficulty level of each activity. The rate at which the level of difficulty is increased is left to the discretion of the teachers who may choose to accelerate children as required. Teachers may also choose to group children according to different skill levels and provide the more advanced children with more challenging skills.

The *Animal Fun *program, consists of the following modules:

Module 1: Body Management (Static balance, Dynamic balance, Climbing)

Module 2: Locomotion (Walking, Jumping, Hopping, Skipping)

Module 3: Object Control (Throwing, Catching, Kicking)

Module 4: Body Sequencing (Trunk, Limbs)

Module 5: Body and Kinaesthetic Management: Trunk and Upper Limb (Eye hand coordination, Visual-kinaesthetic)

Module 6: Fine Motor Planning

Module 7: Tool Control (Pre-scissor/scissor skills, Paint brush use, Drawing/pre-writing skills)

Module 8: Hand Skills (Individual finger strength, Grip strength, Pincer grip)

Module 9: Social/Emotional Development (Laughter, Identifying and labelling feelings, Breathing, Relaxation)

The program includes 30 minutes each day for 4 days/week, usually with an outdoor session of 15 minutes and an indoor session of 15 minutes per day (but allowing flexibility depending on weather etc). Each session focuses on the development of different skills that are linked in with local education system curriculum requirements. Teachers are encouraged to increase the difficulty level as the school term progresses. The program was designed to run for an entire semester (half a year) but should be implemented for at least 10 weeks.

### Outcome Measures

#### Motor ability

Two assessment tools for motor ability will be used as these tools measure different aspects of motor performance. For example, the Bruininks-Oseretsky Test for Motor Proficiency (BOT-2) provides a measure of muscle strength and bi-lateral coordination in addition to the MABC components of manual dexterity, ball skills and balance.

The BOT-2 [53] is the most widely used motor proficiency test, and is designed to assess important aspects of motor development including both gross motor and fine motor components. It is appropriate for examinees from 4-21 years of age and can be used with normal and developmentally disabled children. The test is standardized and norm referenced. In order to minimize participation burden, the Short Form of the test will be used which includes 14 test activities. The average test-retest reliability for the complete battery is 0.87.

The Movement Assessment Battery for Children-2 (MABC-2)[54] is suitable for children aged 3 to 17 years and comprises 8 tasks, three measuring manual dexterity, 3 measuring aiming and catching and 2 measuring balance. Age norms are used to determine an overall standard score and separate standard scores for each of the 3 sub-tests. In addition to the total score a set of qualitative observations allows the examiner to record the child's performance characteristics during the testing. Minimum value of the test-retest reliability of the original MABC is 0.75 and the inter-tester reliability is 0.70. The MABC has been found to correlate well with other motor skill tests [55].

#### Psychosocial Variables

The Social Skills Rating Scale (SSRS)[56] is a standardised measure of social skills appropriate for children aged between 3 and 18 years old. It has a parent (SSRS-P) and a teacher (SSRS-T) version. Both will be used in the current project. The SSRS-P Elementary Level is a 55 item questionnaire consisting of a social skills scale (cooperation, assertion, responsibility and self control) and a problem behaviour scale (externalizing, internalizing and hyperactivity) [56]. The SSRS-T Elementary Level is a 57 item questionnaire consisting of the same two scales, although it does not include the responsibility subscale. The parents and teachers rate the frequency and importance of the child's social skills/behaviours along a three point Likert scale "(i.e., Never, Sometimes, Very Often)" [57] (p.202). The SSRS-T also rates a child's academic competence using a five point Likert scale (i.e. Lowest 10%, Next Lowest 20%, Middle 40%, Next Highest 20%, Highest 10%). The internal coefficients were 0.90 for self-control, 0.88 for interpersonal skills and 0.84 for verbal assertions [58].

The Strengths and Difficulties Questionnaire (SDQ) [59] is a brief behavioural screening questionnaire designed for children aged 4 to 16 years that contains 25 items covering five clinical scales: hyperactivity/inattention, emotional symptoms, conduct problems, peer relationship problems and prosocial behaviour. Summing all the scores from the scales except the prosocial scale generates a total difficulties score. Internal consistency coefficients (Cronbach's α) ranged from .85 for total difficulties to .64 for peer problems. Goodman reported 2-week test- retest reliability of .96 for the TDS. The SDQ correlates highly with the other similar questionnaires although it was considered more sensitive in detecting inattention and hyperactivity and equally effective in detecting internalizing and externalizing problems, and has adequate discriminant and predictive validity [59].

The Pictorial Scale of Perceived Competence and Acceptance (PSPCSA) [60] is a self report measure of perceived competence (physical and cognitive) and perceived social acceptance (peer and maternal) designed for use on children aged 4 - 7 years. This measure was developed as a downward extension of the Perceived Competence Scale for Children [61]. The tool was standardised on a homogeneous population of 191 young children who were mostly Caucasian (96%) and from middle class backgrounds. The pictorial scale consists of 24 items presented in gender specific booklets based around the four subscales of cognitive competence, physical competence, peer acceptance, and maternal acceptance. The reliability and the validity of the scale have been found to be acceptable and stable over 3 years [62]. The internal consistency reliability of the scale was found to be .89 for children with a mean age of 4.45 years based on a population of 90 preschoolers [60]. The test-retest reliability of the PSPCSA was found to be moderate to good on a population of 24 young children with language delays over an 10 - 22 day period (r = .62 - .81) [63].

#### Anthropometric Variables

In addition to the above measures, each child's weight (in light clothing without shoes) and height will also be recorded at each testing session using standard protocols, in order to determine their body mass index (BMI). BMI will be calculated as weight in kilograms divided by the square of height in metres (weight/height^2^). Age and gender corrected BMI z scores [64] will be used for analysis.

The child's girth will be measured around the waist circumference using the navel as a guide. Two measurements to the nearest 0.1 cm will be taken using a non-stretch dressmaker's tape. If there is more than 0.5 cm variation between the two measurements a third will be taken. In analysis, the average of the two closest measurements will be used [65].

### Covariates

#### Intelligence

The Wechsler Preschool and Primary Scale of Intelligence (WPPSI-III [66] will be used to examine the children's performance and verbal intelligence. The WPPSI-III is a standardised test measuring intelligence in children between the ages of 3 and 7 years. For the purposes of this study only four sub-scales of the WPPSI will be used. Performance IQ will be determined using the scaled scores of the block design and object assembly subscales. Verbal IQ will be determined using the scales scores of the receptive vocabulary and information subscales. The WPPSI-III has been chosen because it is considered one of the most popular tests for measuring intelligence in children of this age [67]. It was also chosen because the materials are child friendly and scoring has been made easier [66]. The internal consistency ranges from 0.83 to 0.96 [66]. The test retest reliability for verbal IQ is 0.90 and for the performance IQ is 0.84 [66].

#### Sex

It is anticipated that approximately equal numbers of boys and girls will be recruited. However, if this is not the case then sex will be used as a covariate in the analyses.

### Procedure

Following the schools randomized allocation to a control or intervention condition, teacher training and the collation of parental consent (child assent), a qualified and trained research team will visit each school to conduct the initial testing in Term 2 or 3 of the school year. The testing environment will vary slightly from school to school depending on space availability and whilst some testing areas may not be ideal in terms of lack of distraction and privacy, it was considered important for children of this young age to be kept in a familiar environment. Children will be assessed using all of the measures and researchers will monitor levels of interest and fatigue. Children will be provided with incentive stickers or stamps upon the conclusion of each measure.

Parent packs will be sent home with each child containing the Parent SSRS and SDQ Questionnaires together with a general demographic data form. These packs will be returned in sealed envelopes to the Research Coordinator via the class teacher. Teacher packs containing the Teacher SSRS and SDQ will also be provided to each class teacher and teaching relief will be provided to allow for the timely completion of these questionnaires.

Follow up testing will be conducted under similar circumstances 6 months later in Term 4 of the school year, and in Term 3 of the following year when the children have progressed into Year 1. The children will be assessed on all measures again except for the WPPSI-III, and parents and teachers will again complete the SSRS and SDQ forms.

Parents will be given the option of receiving a summary report on the findings of the initial testing and, if appropriately notated on the parent consent form, a similar report will be provided to the schools. In order to maintain confidentiality, parent reports will not contain any of the information provided by teachers in their SSRS or SDQ Questionnaires.

### Trial Flow

**Figure 1:**
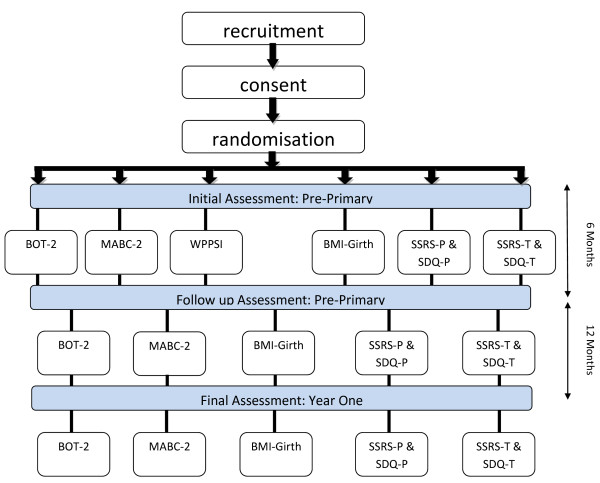
**Methods chart**.

Figure 1 provides an overview of the trial flow.

### Analysis

This is a multivariate nested cohort design [51]. Schools will be randomly allocated to either the intervention or control condition and students will then be followed as a cohort over time to assess the effects of the intervention on a range of dependent variables. The outcomes for individuals can not be assumed to be independent and will be analysed as a Nested Analyses of Covariance (with covariates of sex and IQ) to assess the efficacy of the intervention program (2 groups × 3 testing times). This will be determined by a significant group x time interaction:

• *Hypothesis 1: *The *Animal Fun *program will result in a significantly greater improvement in fine and gross motor scores (measured by MABC-2 and BOT-2) compared with controls when comparing the pre-intervention score with post- intervention score in Pre-Primary and at 1 year follow-up.

• *Hypothesis 2*: The *Animal Fun *program will result in a significantly greater improvement in social skills (measured by SSRS) and perceived competence (measured by PSPCSA) compared with controls when comparing the pre- intervention score with post- intervention scores and at 1 year follow-up.

• *Hypothesis 3*: The Animal Fun program will result in a significantly greater improvement in behavioural problems (measured by SDQ and SSRS) compared with controls when comparing the pre- intervention score with the post- intervention scores and at 1 year follow-up score.

• BMI and girth measurements: These variables will be exploratory.

## Discussion

Appropriate motor skill development is fundamental to normal physical and psychosocial development. Furthermore, poor motor skills have been linked with childhood obesity and poorer mental health outcomes such as lower self worth and higher levels of anxiety and depression. These problems have been found to emerge early in a child's development with some evidence suggesting that they are present as early as 4 years of age. Yet this area of a child's development appears to have considerably less attention in the school setting than other aspects of development such as cognitive and language development. If the intervention to be assessed in this RCT that targets young children before they begin their first year of school is successful, then it may be able to have a considerable impact on the child's early school experience. It may retard or stop the vicious cycle of poor motor ability linking with poor psychosocial outcomes, which then lead to withdrawal from activities that promote physical activity.

Furthermore, if proven successful, this program could be trialed internationally. Given that the theme is animals, it is quite possible that this program could be easily modified for different cultures. Also, it may be possible to progress the program to higher years of schooling to ensure that appropriate motor skills are practiced and maintained throughout the child's school years.

## Competing interests

The authors declare that they have no competing interests.

## Authors' contributions

All authors have contributed substantially to the development of this protocol. In addition, JPP conceived the study and the initial idea for the Animal Fun program. LMS, LJ,AD, CRoberts, CReid, RR, TP, GB and SE all contributed to the development of the Animal Fun program, and all authors have contributed to the study design and manuscript. All authors read and approved the final manuscript.

## Pre-publication history

The pre-publication history for this paper can be accessed here:

http://www.biomedcentral.com/1471-2431/10/78/prepub
